# Novidades e Reflexões sobre o Tratamento Farmacológico da Insuficiência Cardíaca com Fração de Ejeção Preservada

**DOI:** 10.36660/abc.20210753

**Published:** 2022-09-06

**Authors:** Eduardo Thadeu de Oliveira Correia, Evandro Tinoco Mesquita

**Affiliations:** 1 Hospital Universitário Antônio Pedro Niterói RJ Brasil Hospital Universitário Antônio Pedro , Niterói , RJ – Brasil; 2 Complexo Hospitalar de Niterói Niterói RJ Brasil Complexo Hospitalar de Niterói , Niterói , RJ – Brasil

**Keywords:** Insuficiência Cardíaca, Volume Sistólico/efeito de drogas, Inibidores de Enzima Conversora de Angiotensina, Mineralocorticoides, Antagonista Adrenérgicos Beta, Digoxina

## Introdução

Os fenótipos da insuficiência cardíaca (IC) podem ser divididos em categorias de acordo com a fração de ejeção (FE) do ventrículo esquerdo – IC com FE preservada (ICFEp; FE ≥ 50%); IC com FE levemente reduzida (ICFElr; FE 41-49%) e IC com FE reduzida (ICFEr; FE≤40%). ^1^ No entanto, as diferenças entre os fenótipos da IC vão além da FE. Enquanto a ICFEp desenvolve-se a partir de uma interação de comorbidades que leva à doença cardíaca estrutural e sintomas da IC, a ICFEr geralmente desenvolve-se devido a um insulto cardíaco que reduz o débito cardíaco. ^1 , 2^ Além disso, embora múltiplas terapias possam melhorar o prognóstico da ICFEr, somente os inibidores do cotransportador de sódio-glicose-2 (SGLT2i) melhoraram os desfechos da ICFEp, demonstrado em um ensaio controlado randomizado (ECR). ^3^ Nesta carta científica, avaliamos evidências de terapias farmacológicas com potencial benefício na ICFEp.

### Tratamento das etiologias da ICFEp e condições associadas

O manejo das etiologias da ICFEp e comorbidades (p.ex., hipertensão, diabetes, doença arterial coronariana, obesidade, anemia, doença renal crônica) é essencial para evitar a progressão da doença e reduzir hospitalização. ^1^ Pacientes com cardiomiopatia causada por amiloidose por transtirretina também se beneficiam de tafamidis, que reduziu em 30% e 32% o risco de mortalidade por todas as causas e internações por doenças cardiovasculares, respectivamente, em comparação a placebo. ^4^

### Inibidores da enzima conversora de angiotensina (IECA), bloqueadores de receptor de angiotensina (BRAs), e inibidor da neprilisina e do receptor da angiotensina

ECRs anteriores, tais como o PEP-CHF, ^5^ o CHARM-Preserved ^6^ e o I-PRESERVE ^7^ não mostraram benefício significativo dos IECA ou BRAs em pacientes com ICFEp. Khan et al., ^8^ confirmaram esses achados, mas demonstraram, em uma análise agrupada dos ECRs, uma tendência de menor risco de hospitalização por IC. ^8^ Em seguida, sacubitril/valsartana surgiu como um medicamento promissor para melhorar desfechos na ICFEp, mas falhou em demonstrar benefício em seu desfecho primário de hospitalização por IC ou morte cardiovascular no ensaio PARAGON-HF. ^9^ No entanto, mulheres com ICFEp podem se beneficiar do sacubitril/valsartana, uma vez que o fármaco reduziu em 27% o desfecho primário em comparação ao placebo, em uma análise de subgrupo pré-especificada. ^9^ Evidências de uma meta-análise de ECRs mostrou que o sacubitril/valsartana levou à redução de níveis de NT-pro-BNP e melhora da qualidade de vida de pacientes com ICFEp. ^10^ Assim, pode-se preferir sacubitril/valsartana a BRAs ou IECA em pacientes com indicação para inibidores do sistema renina-angiotensina-aldosterona devido a comorbidades.

### Antagonista dos receptores mineralocorticoides (ARMs)

No ensaio TOPCAT, a espironolactona não reduziu o desfecho primário de morte cardiovascular, parada cardíaca abortada ou hospitalização por IC em pacientes com ICFEp em comparação a placebo, embora tenha sido eficaz em pacientes como níveis elevados de peptídeos natriuréticos. ^11 , 12^ Enquanto pacientes nas Américas tiveram uma redução de 18% no risco do desfecho primário com a espironolactona, pacientes na Rússia e na Geórgia não tiveram melhora no prognóstico com a medicação. ^11^ Tal fato pode ser explicado por diferenças na randomização, pacientes que não tomaram a medicação, e taxas de eventos mais baixas na Rússia e na Geórgia. ^11 , 13^ Evidências de uma meta-análise mostraram que a espironolactona reduziu internações, melhorou a classe funcional da *New York Heart Association* (NYHA) e reduziu níveis do peptídeo natriurético tipo-B em pacientes com ICFEp. ^14^

### Diuréticos

Devido a questões éticas em se realizar ECRs para o uso de diuréticos, seus efeitos sobre o prognóstico em longo prazo na ICFEp são desconhecidos. No entanto, uma análise pós-hoc do ensaio CHAMPION mostrou que ajustes na terapia com diuréticos e vasodilatadores de acordo com a pressão arterial pulmonar reduziu em 46% a razão de incidência de hospitalização por IC em pacientes com ICFEp classe III da NYHA. ^15^ Isso reforça a necessidade de se controlar edema pulmonar e periférico, e indica que diuréticos não só controlam sintomas como também reduzem internações por IC.

### Inibidores do cotransportador de sódio-glicose-2 (SGLT2i)

Dados do ensaio EMPEROR-Preserved mostrou que a empagliflozina reduziu o risco de desfecho primário de morte cardiovascular ou hospitalização por IC em pacientes com ICEFp em comparação a placebo. ^3^ Em uma análise exploratória, a empagliflozina também reduziu internações por IC que requeriam cuidado intensivo, internações que requeriam droga vasopressora ou inotrópicos positivos, e a necessidade de se intensificar terapia com diuréticos em pacientes ambulatoriais. ^16^ Ainda, a probabilidade de melhora na classe funcional da NYHA foi maior nos pacientes que receberam empagliflozina. ^16^

### Betabloqueadores e outras terapias

**Figura 1:**
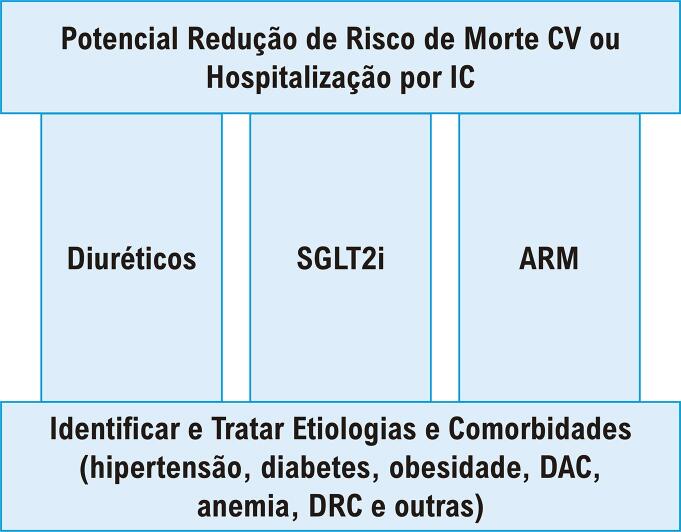
Proposta de terapia tripla para insuficiência cardíaca com fração de ejeção preservada. DAC: doença arterial coronariana; DRC: doença renal crônica; CV: cardiovascular; IC: insuficiência cardíaca; ARMs: antagonistas dos receptores mineralocorticoides; SGLT2i: inibidores do cotransportador de sódio-glicose-2; somente a empagliflozina tem evidências de um ensaio randomizado robusto. 3 Análises pós-hoc dos ensaios CHAMPION e TOPCAT podem corroborar o uso de diuréticos e ARMs. 11-13,15

Em uma meta-análise de ECRs, os betabloqueadores não reduziram o risco de mortalidade por todas as causas ou morte cardiovascular em pacientes com ICFEp em ritmo sinusal ou com fibrilação atrial. ^17^ Tanto a digoxina como terapias cujo alvo era a via do óxido nítrico/monofosfato cíclico de guanosina não mostraram sucesso na melhora dos desfechos na ICFEp. ^1 , 18^ Detalhes de ECRs de fase III que investigaram tratamentos farmacológicos em pacientes com ICFEp estão descritos na Tabela 1 . Após rever as evidências aqui descritas, nós delineamos uma proposta de terapia tripla com o potencial de melhorar os desfechos de pacientes com ICFEp, ilustrada na Figura 1 .

**Tabela 1:** Ensaios controlados randomizados de fase III de terapias farmacológicas para insuficiência cardíaca com fração de ejeção preservada

Estudo	Droga	Critérios de inclusão	Mortalidade por todas as causas	Mortalidade cardiovascular	Mortalidade cardiovascular ou hospitalização por IC	Hospitalização por IC
PEP-CHF ^5^	Perindopril	Índice de motilidade da parede do VE ≥ 1,4, IC sintomática tratada com diuréticos, disfunção diastólica, idade ≥ 70 anos	HR: 1,09 (0,75-1,58)	HR: 0,98 (0,63-1,53)	NR	HR: 0,86 (0,61-1,20)
CHARM-Preserved ^6^	Candesartana	FEVE > 40%, NYHA II-IV, História de internação por doença cardiovascular	NR	HR: 0,99 (0,80-1,22)	HR: 0,89 (0,77-1,03)	HR: 0,85 (0,72-1,01)
I-PRESERVE ^7^	Irbesartana	FEVE ≥ 45%, NYHA III-IV ou NYHA II com internação por IC nos últimos seis meses, idade ≥ 60 anos	HR: 1,00 (0,88-1,14)	HR: 1,01 (0,86-1,18)	HR: 0,96 (0,84-1,09)	HR: 0,95 (0,81-1,10)
PARAGON-HF ^9^	Sacubitril-Valsartana	IC com FEVE ≥ 45%, NYHA II-IV, aumento do átrio esquerdo ou hipertrofia do VE e BNP ≥ 300 pg/mL ou NT-proBNP ≥ 900 pg/mL ou internação por IC nos últimos nove meses	HR: 0,97 (0,84-1,13)	HR: 0,95 (0,79-1,16)	RaR: 0,87 (0,75–1,01)	RaR: 0,85 (0,72-1,00)
TOPCAT ^11^	Espironolactona	FEVE ≥ 45%, ≥ 1 sinal de IC e ≥ um sintoma de IC, internação por IC nos últimos nove meses, ou BNP ≥ 100 pg/mL, NT-proBNP ≥ 360 pg/mL, idade ≥ 50 anos	HR: 0,91 (0,77-1,08)	HR: 0,90 (0,73-1,12)	HR: 0,89 (0,77-1,04)	**HR: 0,83 (0,69-0,99)**
EMPEROR-Preserved ^3^	Empagliflozina	IC com FEVE ≥ 40%, NYHA II-IV, idade ≥18 anos, NT-proBNP > 300 pg/mL ou NT-proBNP > 900 pg/mL para pacientes com IC e FA	HR: 1,00 (0,87-1,15)	HR: 0,91 (0,76-1,09)	**HR: 0,79 (0,69-0,90)**	**HR: 0,73 (0,61-0,88)**
DIG-PEF ^18^	Digoxina	IC com FEVE > 45%, RS	RiR: 0,99 (0,76-1,28)	RiR: 1,00 (0,73-1,36)	RiR: 0,88 (0,70-1,11)	RiR: 0,79 (0,59-1,04)

*FA: fibrilação atrial; VE: ventrículo esquerdo; IC: insuficiência cardíaca; FEVE: fração de ejeção do ventrículo esquerdo; HR: hazard ratio; NT-proBNP: fragmento N-terminal do peptídeo natriurético tipo B; NYHA: New York Heart Association; RaR: rate ratio; RiR: risk ratio; RS: ritmo sinusal; NR: não reportado.*

## Conclusões

Até o momento, a empagliflozina é a única terapia farmacológica com dados robustos de randomização que apoiem seus benefícios na ICFEp. Contudo, como discutido acima, uma combinação de diuréticos, ARMs e SGLT2i pode reduzir a mortalidade e a hospitalização em pacientes com ICFEp. Serão necessários outros ECRs investigando novas terapias para a ECFEp.
