# Supplementary material for: Novidades e Reflexões sobre o Tratamento Farmacológico da Insuficiência Cardíaca com Fração de Ejeção Preservada
Source: Arq Bras Cardiol. 2022 Sep 6;119(4):627–30. [Article in Portuguese] doi: 10.36660/abc.20210753 (PMC9563882; doi:10.36660/abc.20210753)
Supplement: Translation

# Translation: New Insights into Medical Therapy for Heart Failure with Preserved Ejection Fraction

**Authors:** Eduardo Thadeu de Oliveira Correia, Evandro Tinoco Mesquita

**Affiliations:** 1 Brazil Hospital Universitário Antônio Pedro, Niterói, RJ – Brazil; 2 Brazil Complexo Hospitalar de Niterói, Niterói, RJ – Brazil

**Keywords:** Heart Failure, Stroke Volume/drug effects, Angiotensin-Converting Enzyme Inhibitors, Mineralocoirticoids, Receptor Antagonists, Digoxin

## Introduction

Heart failure (HF) phenotypes can be divided into categories according to the left ventricular ejection fraction (EF) – HF with preserved EF (HFpEF; EF ≥ 50%); HF with mildly reduced EF (HFmrEF; EF 41-49%) and HF with reduced EF (HFrEF; EF ≤ 40%).^1^ However, HF phenotypes differ beyond just a different EF. While HFpEF develops from an interplay of comorbidities that lead to structural heart disease and HF symptoms, HFrEF usually develops due to a cardiac insult that reduces cardiac output.^1 , 2^ Moreover, while multiple therapies can improve the prognosis of HFrEF, only sodium-glucose cotransporter 2 inhibitors (SGLT2i) improved outcomes in HFpEF in a randomized controlled trial (RCT).^3^ In this letter, we explore evidence for medical therapies that could benefit HFpEF patients.

### Treatment of HFpEF etiologies and associated conditions

The management of HFpEF etiologies and comorbidities (e.g., hypertension, diabetes, coronary artery disease, obesity, anemia, chronic kidney disease) is essential to avoid disease progression and reduce hospitalization.^1^ Patients with transthyretin amyloid cardiomyopathy also benefit from tafamidis, that reduced by 30% and 32% the risk for all-cause mortality and cardiovascular (CV) hospitalizations, respectively, compared with placebo.^4^

### Angiotensin-converting-enzyme inhibitors (ACEi), angiotensin receptor blockers (ARBs) and angiotensin receptor-neprilysin Inhibitors

Previous RCTs, such as the PEP-CHF,^5^ CHARM-Preserved^6^ and I-PRESERVE^7^ showed no significant benefit of ACEi or ARBs in HFpEF patients. Khan et al.^8^ confirmed these findings but showed, in a pooled analysis of RCTs, a trend towards reduced HF hospitalization risk.^8^ Subsequently, sacubitril/valsartan has emerged as a promise to improve outcomes in HFpEF, but failed to meet its primary endpoint of HF hospitalization or CV death in the PARAGON-HF trial.^9^ Nonetheless, women with HFpEF may benefit from sacubitril/valsartan, since it reduced by 27% the primary outcome compared with placebo in a prespecified subgroup analysis.^9^ Evidence from a meta-analysis of RCTs showed that sacubitril/valsartan led to reductions in NT-proBNP and improvements in quality of life in HFpEF patients.^10^ Hence, sacubitril/valsartan could be preferred over ARBs or ACEi in patients with indications for renin-angiotensin system inhibitors due to comorbidities.

### Mineralocorticoid receptor antagonists (MRAs)

In the TOPCAT trial, spironolactone did not reduce the primary outcome of CV death, aborted cardiac arrest or HF hospitalization in HFpEF patients compared with placebo, although it was effective among patients with elevated natriuretic peptides.^11 , 12^ Surprisingly, while patients in the Americas experienced an 18% risk reduction of the primary outcome, in Russia and Georgia, spironolactone did not improve prognosis.^11^ This can be explained by differences in randomization, patients that did not take the drug, and lower event rates in Russia and Georgia.^11 , 13^ Additional evidence from a meta-analysis showed that spironolactone reduced hospitalizations, improved New York Heart Association (NYHA) class and decreased levels of b-type natriuretic peptide in HFpEF patients.^14^

### Diuretics

Due to ethical issues in conducting RCTs for diuretic use, their effects on long-term prognosis in HFpEF are unknown. However, a post-hoc analysis of the CHAMPION trial showed that changes in diuretic and vasodilator therapies according to pulmonary artery pressure reduced by 46% the incidence rate ratio of HF hospitalization in HFpEF with NYHA class III.^15^ This reinforces the need of controlling peripheral and pulmonary edema and indicates that diuretics not only control HF symptoms but may also reduce HF hospitalization.

### Sodium–glucose cotransporter 2 inhibitors (SGLT2i)

Data from the EMPEROR-Preserved trial showed empagliflozin reduced the risk for the primary endpoint of CV death or HF hospitalization in HFpEF patients compared with placebo.^3^ In an exploratory analysis, empagliflozin also reduced HF hospitalizations that required intensive care, hospitalizations that required vasopressors or positive inotropes, and the need for diuretic intensification in outpatients.^16^ Moreover, patients assigned to empagliflozin were more likely to have improved NYHA class.^16^

### Beta-blockers and other therapies

In a meta-analysis of RCTs, beta-blockers did not reduce the risk for all-cause mortality or CV death in HFpEF patients with sinus rhythm or atrial fibrillation.^17^ Digoxin and therapies targeting the nitric oxide-cyclic guanosine monophosphate pathway also failed to improve endpoints in HFpEF.^1 , 18^ Phase III RCTs that investigated pharmacological therapies in HFpEF patients are detailed in Table 1 . After reviewing the evidence presented above, we outlined a triple therapy proposal with potential to improve outcomes of HFpEF patients, that is illustrated in Figure 1 .

**Table 1:** Phase III randomized controlled trials of pharmacological therapies in heart failure with preserved ejection fraction

Study	Drug	Inclusion Criteria	All-Cause Mortality	CV Mortality	CV Death or HF Hospitalization	HF Hospitalization
PEP-CHF^5^	Perindopril	LV wall motion index ≥ 1.4, symptomatic HF treated with diuretic, diastolic dysfunction, age ≥ 70 years	HR: 1.09 (0.75-1.58)	HR: 0.98 (0.63-1.53)	NR	HR: 0.86 (0.61-1.20)
CHARM-Preserved^6^	Candesartan	LVEF > 40%, NYHA II-IV, history of CV hospitalization	NR	HR: 0.99 (0.80-1.22)	HR: 0.89 (0.77-1.03)	HR: 0.85 (0.72-1.01)
I-PRESERVE^7^	Irbesartan	LVEF ≥ 45%, NYHA III-IV or NYHA II with HF hospitalization in the past 6 months, age ≥ 60 years	HR: 1.00 (0.88-1.14)	HR: 1.01 (0.86-1.18)	HR: 0.96 (0.84-1.09)	HR: 0.95 (0.81-1.10)
PARAGON-HF^9^	Sacubitril-Valsartan	HF with LVEF ≥ 45%, NYHA II-IV, left atrial enlargement or LV hypertrophy and BNP ≥ 300 pg/mL or NT-proBNP ≥ 900 pg/mL or HF hospitalization in the last 9 months	HR: 0.97 (0.84-1.13)	HR: 0.95 (0.79-1.16)	RaR: 0.87 (0.75–1.01)	RaR: 0.85 (0.72-1.00)
TOPCAT^11^	Spironolactone	LVEF ≥ 45%, ≥ 1 HF sign and ≥ 1 HF symptom, HF hospitalization within the past 12 months, or BNP ≥ 100 pg/mL or NT-proBNP ≥ 360 pg/mL, age ≥ 50 years	HR: 0.91 (0.77-1.08)	HR: 0.90 (0.73-1.12)	HR: 0.89 (0.77-1.04)	**HR: 0.83 (0.69-0.99)**
EMPEROR-Preserved^3^	Empagliflozin	HF with LVEF ≥ 40%, NYHA II-IV, age ≥ 18 years, NT-proBNP > 300 pg/mL or NT-proBNP > 900 pg/mL for patients with HF and AF	HR: 1.00 (0.87-1.15)	HR: 0.91 (0.76-1.09)	**HR: 0.79 (0.69-0.90)**	**HR: 0.73 (0.61-0.88)**
DIG-PEF^18^	Digoxin	HF with LVEF > 45%, SR	RiR: 0.99 (0.76-1.28)	RiR: 1.00 (0.73-1.36)	RiR: 0.88 (0.70-1.11)	RiR: 0.79 (0.59-1.04)

*AF: atrial fibrillation; CV: cardiovascular; HF: heart failure; HR: hazard ratio; LV: left ventricular; LVEF: left ventricular ejection fraction; NT-proBNP: N-terminal-pro hormone b-type natriuretic peptide; NYHA: New York Heart Association; RaR: rate ratio; RiR: risk ratio; SR: sinus rhythm; NR: not reported.*

**Figure:** A Triple Therapy Proposal for Heart Failure with Preserved Ejection Fraction. CAD: coronary artery disease; CKD: chronic kidney disease; CV: cardiovascular; HF: heart failure; MRA: mineralocorticoid receptor antagonist; SGLT2i: sodium-glucose 2 cotransporter inhibitor. Only empagliflozin has evidence from a robust randomized trial. 3 Post-hoc analyses of the CHAMPION and TOPCAT trials may support the use of diuretics and MRA. 11-13,15

## Conclusions

Empagliflozin is the only pharmacological therapy with robust randomized data to support its benefit in HFpEF to this date. However, as discussed above, a combination of diuretics, MRAs and SGLT2i may reduce mortality and hospitalization in HFpEF. Future RCTs investigating novel therapies for HFpEF are needed.
